# Computational Comparison of the Mechanical Behavior of Aortic Stent-Grafts Derived from Auxetic Unit Cells

**DOI:** 10.1007/s13239-023-00706-x

**Published:** 2023-12-18

**Authors:** Rahul Vellaparambil, Woo-Suck Han, Pierluigi Di Giovanni, Stéphane Avril

**Affiliations:** 1 Mines Saint-Etienne, Université Jean Monnet Saint-Etienne, INSERM, SAINBIOSE U1059, 42023 Saint-Etienne, France; 2 Research and Development Department, HSL S.R.L, Trento, Italy

**Keywords:** Aortic stent-graft, Auxetic stent, Finite element analysis, Mechanical behavior, Stent design, Aortic aneurysm, Z-stent, Circular stent

## Abstract

**Purpose:**

Inappropriate stent-graft (SG) flexibility has been frequently associated with endovascular aortic repair (EVAR) complications such as endoleaks, kinks, and SG migration, especially in tortuous arteries. Stents derived from auxetic unit cells have shown some potential to address these issues as they offer an optimum trade-off between radial stiffness and bending flexibility.

**Methods:**

In this study, we utilized an established finite element (FE)-based approach to replicate the mechanical response of a SG iliac limb derived from auxetic unit cells in a virtual tortuous iliac aneurysm using a combination of a 180° U-bend and intraluminal pressurization. This study aimed to compare the mechanical performance (flexibility and durability) of SG limbs derived from auxetic unit cells and two commercial SG limbs (Z-stented SG and circular-stented SG models) in a virtual tortuous iliac aneurysm. Maximal graft strain and maximum stress in stents were employed as criteria to estimate the durability of SGs, whereas the maximal luminal reduction rate and the bending stiffness were used to assess the flexibility of the SGs.

**Results:**

SG limbs derived from auxetic unit cells demonstrated low luminal reduction (range 4–12%) with no kink, in contrast to Z-stented SG, which had a kink in its central area alongside a high luminal reduction (44%).

**Conclusions:**

SG limbs derived from auxetic unit cells show great promise for EVAR applications even at high angulations such as 180°, with acceptable levels of durability and flexibility.

**Supplementary Information:**

The online version contains supplementary material available at 10.1007/s13239-023-00706-x.

## Introduction

In 1991, Parodi et al. [1] pioneered the field of endovascular aortic repair (EVAR) by deploying a stent-graft (SG) into an abdominal aortic aneurysm (AAA) to reestablish blood flow, effectively excluding the AAA. When compared to conventional surgical repair, EVAR has demonstrated lower short-term mortality and complication rates [2]. Nonetheless, EVAR has been associated with a notable proportion of re-interventions, typically ranging between 20 to 30%, primarily due to issues like endoleaks, SG migration, and SG component degradation [3, 4]. Furthermore, presently available SGs have been reported to have durability concerns [5], along with complications like endoleaks, SG migration, and SG component disintegration [6–8]. The limited flexibility of SGs has been linked to the development of kinks in tortuous iliac arteries [9, 10] and other complications in tortuous AAAs [11, 12].

In the past two decades, there have been limited publications investigating the interplay between the mechanical properties of SGs and stent designs, specifically concerning factors related to flexibility and durability. It is widely acknowledged that Kleinstreuer et al. [13] stands as the inaugural study to employ the finite element method (FEM) to assess the mechanical attributes of a SG, utilizing a stent with a diamond lattice design. Demanget et al. [14] conducted a comprehensive analysis of the flexibility of two commercially available SGs: one with a Z-stent design and the other with a spiral-stent design. Their numerical investigation based on FEM revealed that the spiral-stented SGs exhibited superior flexibility when compared to the Z-stented SGs. These numerical findings were rigorously corroborated through experimental testing carried out by Demanget et al. [15]. Subsequently, the same methodology detailed in Demanget et al. [14] was applied to evaluate the flexibility and durability of eight different commercially available SGs, as documented in Demanget et al. [16].

In our recent investigation [17], we explored the potential of employing re-entrant (RE), chiral re-entrant (CRE), and chiral star-based (CS) auxetic stent designs, specifically focusing on their radial stiffness and flexibility attributes for applications in EVAR. These unique designs were subject to a comprehensive comparison with the traditional diamond lattice design described in Kleinstreuer et al. [13] using a series of numerical bench tests. The results of these tests have already revealed intriguing advantages of auxetic stents over the conventional diamond stent design, particularly in terms of their enhanced flexibility.

Building upon these promising findings, this study involves an in-depth examination of the mechanical behavior of novel SG candidates incorporating stents based on the aforementioned auxetic unit cells. These stents will be seamlessly integrated with a polymer graft, and we will utilize a numerical model to apply boundary conditions that replicate the real-world deformations that a SG would experience within a highly tortuous aortoiliac aneurysm, as previously delineated in [16]. Our assessment of these SG candidates will encompass a thorough evaluation of their flexibility and durability attributes, following the relevant methodology as outlined in [16]. Our primary objective is to identify SG candidates derived from auxetic unit cells that can emulate or surpass the simulated performance of a commercially available SG using circular or Z-stent unit cells in a comparable tortuous setting.

## Materials and Methods

### FEA Methodology For Modeling SG

A three-step-based boundary condition was implemented to predict the in vivo deformations suffered by a SG in a tortuous iliac aneurysm. For consistency and benchmarking purposes, the Zenith Low Profile (Zenith-LP) Z-stented SG, (Cook Medical Europe, Bjaeverskov, Denmark), was chosen as the reference template against which all other designs in this study were compared.

All individual numerical simulations were run using an explicit solver on the Abaqus v2020 finite element solver (Dassault Systemes Simulia Corp. Providence, RI, USA) with a total running time of 22 hours based on an usage of eight cores on the high-performance computing cluster of Mines Saint-Etienne (total capacity: 11 Tflops with 26 nodes, summing up to 384 cores and 1 TB of RAM). To enforce a quasi-static simulation, we utilized a solver setting concerning the mass-scaling technique on Abaqus with a stable target time increment of 5 × 10^−8^ s based on a sensitivity analysis. We ensured that the ratio between the global kinetic and internal energies was maintained below a limit of 5% for all numerical simulations [16, 17].

### Stent Modeling

All stents were discretized using 2-node linear B31 beam elements [14, 18, 19] as a nitinol wire with 0.34 mm diameter on Abaqus. Nitinol was modeled as a superelastic material in Abaqus, and its constituent parameters are displayed in Table S1 referenced in supplementary material. Self-contact between stent sections was allowed for all numerical simulations.

### Graft and Suture Modeling

The grafts are modeled as cylindrical structures discretized with 4-node quadrilateral shell S4R elements with reduced integration. A shell model was implemented in Abaqus to represent the dacron/polyethylene terephthalate (PET) graft with modified bending rigidity attributes in circumferential and longitudinal directions obtained by Demanget et al. [15]. The constituent parameters of the grafts are listed in Table S2 mentioned in supplementary material. Stents are generally combined with the graft using surgical sutures, which is modeled using a “tie” constraint in Abaqus that enforces the kinematic connection between the outer surface of graft and stent to restrict any sliding or separation between the two SG components. Self-contact between the graft sections was considered for all numerical simulations.

### Geometry and Mesh

In this investigation, we created numerical models for SG candidates, placing a specific emphasis on the iliac limbs of these SGs. This focus on iliac limbs is warranted due to the substantial deformations that iliac arteries commonly experience both before and after they are subjected to EVAR procedures, largely owing to their inherently tortuous nature [14]. The study takes into consideration the geometric properties of two commercially available SG limbs: Zenith-LP and Aorfix (Lombard Medical, Didcot, UK) Detailed geometric information about these limbs can be found in Tables S3 and S4 in the supplementary material. To ensure mesh density convergence for our SG finite element model, we gathered data on maximal stress in the stent, graft strains, and maximal reaction moment magnitudes similar to the mesh convergence criteria employed by Liu et al. [20]. Based on the outcomes of this mesh convergence test, we selected an element size of 0.5 mm for the stent and 0.3 mm for the graft. The resultant meshes for all the numerical models of the SG candidates featured in this study are presented in Table 1.

**Table 1:** Mesh features of SG candidates considered in this study

SG Type	Stent Elements	Graft Elements
AO-LP	2222	50,840
Zenith-LP	1440	50,840
ZLP8	2432	50,840
RE-UL	3968	50,840
CRE-UL	4224	50,840
CS-UL	3968	50,840
RE-DIA	3840	50,840
CRE-DIA	3904	50,840
CS-DIA	3776	50,840

### Conventional SG Geometries

For this study, we chose two commercial SG designs, Zenith-LP and Aorfix, as previously utilized in the numerical models described in Demanget et al. [16]. To enhance the potential for SG stent design optimization, the number of peaks encircling the Zenith Low Profile SG's circumference has been increased from the original four to eight. This modified model is hereafter denoted as Zenith Low Profile-8 (ZLP8). Additionally, given that spiral-stented SGs like Aorfix exhibit superior flexibility when compared to Z-stent-type SGs [14, 15], we developed an additional SG design based on the ZLP8 template. In this new design, the original Z-stent rings are replaced with circular stents, resulting in a configuration referred to in this study as AO-LP.

### Auxetic SG Geometries

**Fig. 1:**
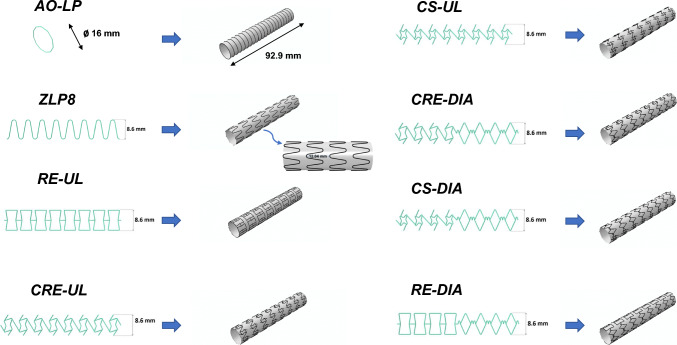
Compilation of all SG designs considered in this study and their numerical models

In our previous study [17], we presented the advantages of three different auxetic stent designs (re-entrant or RE, chiral-re-entrant hybrid or CRE, and chiral-star or CS) in the context of EVAR applications, focusing on their radial stiffness and bending flexibility characteristics. All three of these auxetic stent designs exhibited superior flexibility when compared to the conventional diamond stent design [17]. Consequently, these three auxetic designs were included in this current study. Since most conventional SG designs consist of stent rings that are not interconnected, we recreated all auxetic SG designs using the standard ZLP8 template without any interconnections, resulting in re-entrant-unlinked (RE-UL), chiral-re-entrant-unlinked (CRE-UL), and chiral-star-unlinked (CS-UL) configurations, as depicted in Fig. 1.

Inspired by the work of Han and Lu [21], which demonstrated that a non-uniform Poisson's ratio distribution (NUPR) in stent designs, combining negative Poisson's ratio (NPR) and positive Poisson's ratio (PPR), could be optimized for use in curved arteries, we decided to incorporate a diamond cell with a PPR [17] into the three auxetic stent unit cells. To enhance bending flexibility, we linked these unit cells in a “V” configuration [21], resulting in the creation of the novel CRE-DIA (chiral-re-entrant–diamond), RE-DIA, and CS-DIA candidates, respectively. Additionally, we developed three variations of CRE-DIA to identify the most effective arrangement of auxetic and diamond unit cells based on their mechanical performance after bending and intraluminal pressurization. The details of these variations are provided in the supplementary material.

### Boundary Conditions

**Fig. 2:**
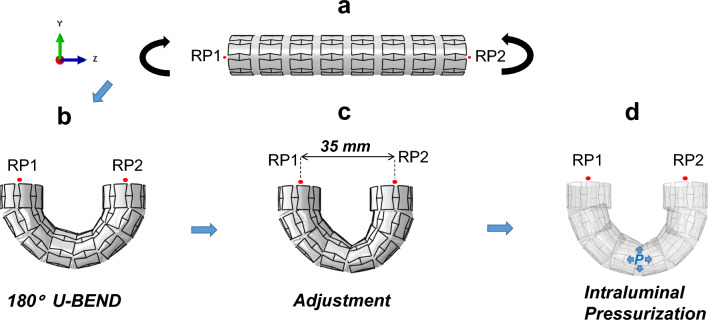
Illustration of the boundary conditions on the SG models in (**a**), followed by the global deformed state of RE-UL during 180° bending in (**b**), stretching condition applied to enforce a 35 mm separation between the reference points in (**c**), and intraluminal pressurization in (**d**). RP1 and RP2 indicate the reference points on which the boundary conditions are enforced in step 2a and 2c

In order to simulate the most extreme angulated scenario that a SG limb might encounter, we initiated the bending phase with a 180° U-Bend loading. This choice was based on the highest angulation observed in the iliac artery [14]. For the final intraluminal pressurization step, we established an ultimate pressure magnitude of 150 mmHg. This value was derived from the maximum systolic blood pressure recorded in hypertensive patients, as noted in Demanget et al. [15]. The rationale behind these test conditions was to assess the performance of a SG under the most challenging conditions. If a SG could withstand this ultimate load without developing kinks, graft fabric tears, or stent fractures, it would be considered suitable for deployment in highly angulated settings. The boundary conditions for studying their mechanical performance were assigned using a three-step method, as defined below:

A. Bending (180°)
● Each SG model endured a 180° bending operation, where both SG ends were represented as rigid bodies driven by reference points (RP1 and RP2), respectively, as shown in Fig. 2a, where the rotation-controlled boundary conditions were applied.
● Opposite rotations are applied at the reference points along the X-axis, as shown in Fig. 2a, with translation along the Z-axis only fixed for RP1, but allowed for RP2 to ameliorate the tension across the SG length in this step. Furthermore, out-of-plane rotations and translations along the other axes (Y and Z) were constrained to prevent rigid body motion.
B. Adjustment (35 mm)
● Post-bending, the separation between RP1 and RP2 is regulated to reach a magnitude of 35 mm along the Z-axis using opposite translations at the reference points; thus, an effective visual comparison can be conducted between different SG candidates, as depicted in Fig. 2c.
C. Intraluminal Pressurization (0.02 MPa)
● Finally, the internal surface of the graft was subjected to a pressure of 150 mmHg (0.02 MPa), as illustrated in Fig. 2d with no other boundary conditions enforced on SG extremities.

### Crimp Radial Force

As an additional measure to distinguish between top-performing SG candidates, a crimp test of a single stent ring was conducted, where the original stent diameter (16 mm) was reduced to 60% using 12 rigid plates [17]. Based on the instructions for use manual for Zenith Flex® AUI AAA Endovascular Graft with the Z-Trak™ Introduction System [22] for an Iliac Leg Graft with a 16 mm diameter, the appropriate outer diameter of the introducer sheath should be 16Fr or 6.0 mm, resulting in a reduction in diameter of approximately 62.5%. Since the Zenith SG serves as the foundational design for all auxetic SGs, we chose to implement a 60% reduction in the diameter of each individual stent ring to replicate realistic crimp-related stress and strain conditions for performance assessment. These plates were radially patterned across the stent ring to enforce radial compression using compressive displacement boundary conditions until a stent diameter magnitude of 6.4 mm was achieved. After that the stents were released from radial compression where the plates return to their original position. A penalty friction coefficient of 0.2 magnitude was defined in the tangential direction with self-contact between stent wires, included in the contact definition in Abaqus [17].

### Assessment Criteria

We applied the same criteria as Demanget et al. [16], where the resilience and adaptability characteristics of all SG candidates were documented and assessed based on the aforementioned boundary conditions.

### Luminal Reduction Rate (LR)

The LR criterion refers to the reduction of SG cross-sectional area between the nominal state ${S}_{o}$ and deformed state S at the end of simulation post bending and pressurization as referenced in Eq. 1, where

1 $${S}_{o}= \pi {R}_{G}^{2}\;({R}_{G}\text{ refers to radius of graft})$$

1 $$\text{LR }= 100 \left(1- \frac{S}{{S}_{o}}\right)$$

A ${LR}_{max}$ magnitude exceeding 60–70% may be conducive to propagating SG thrombosis as per feedback from surgical experts [14, 16], hence a critical clinical limit of 60% [16] for ${LR}_{max}$ will be utilized in this work. Procurement of the deformed SG cross-sectional area was performed with cut planes on META Post-processor v22.0 (BETA CAE System, Switzerland), following which the calculation of the SG cross-sectional area was done by using ANSA Pre-processor v22.0 (BETA CAE System, Switzerland).

### Torque for bending (TRB)

The TRB was evaluated using the collection of reaction moments derived from reference points RP1 and RP2 at the end of the bending simulation.

### Stresses in Stent $({\sigma }_{S}^{max})$

The stress magnitude of the stents was recorded using the maximal Von Mises stresses derived from Abaqus at the end of the simulation post-bending and pressurization.

### Strains in Graft $({\varepsilon }_{CG}$ and ${\varepsilon }_{LG})$

Circumferential logarithmic strain ${\varepsilon }_{CG}$ and longitudinal logarithmic strains ${\varepsilon }_{LG}$ located on the graft were extracted from Abaqus to evaluate the durability of the graft at the end of numerical simulation (post-bending and pressurization). Ultimate strain limits for the graft are referenced in table S2, which will be utilized as a critical limit during the assessment. If these strain limit thresholds were crossed at the end of a simulation, we could make the claim that the SG candidate would potentially suffer fabric tears in a highly angulated setting.

### Crimp Test: Principal Strain and Force–Diameter Curves

The radial force from the crimp test was compiled using the sum of the 12 radial reactive forces generated from the 12 plates to obtain the force vs. diameter curves for the top-performing SG candidates. In addition, the maximal principal crimp strain for the selected SG candidates was extracted from Abaqus during the crimp test to examine whether any SG candidate has strains exceeding the critical limit of 12% for Nitinol based stents [13]. If the crimp strain exceeded the critical limit, we could claim that there was a high chance of SG stent-fracture occurring for the SG candidate in a highly angulated setting.

## Results

### Test Case: Zenith LP SG

**Fig. 3:**
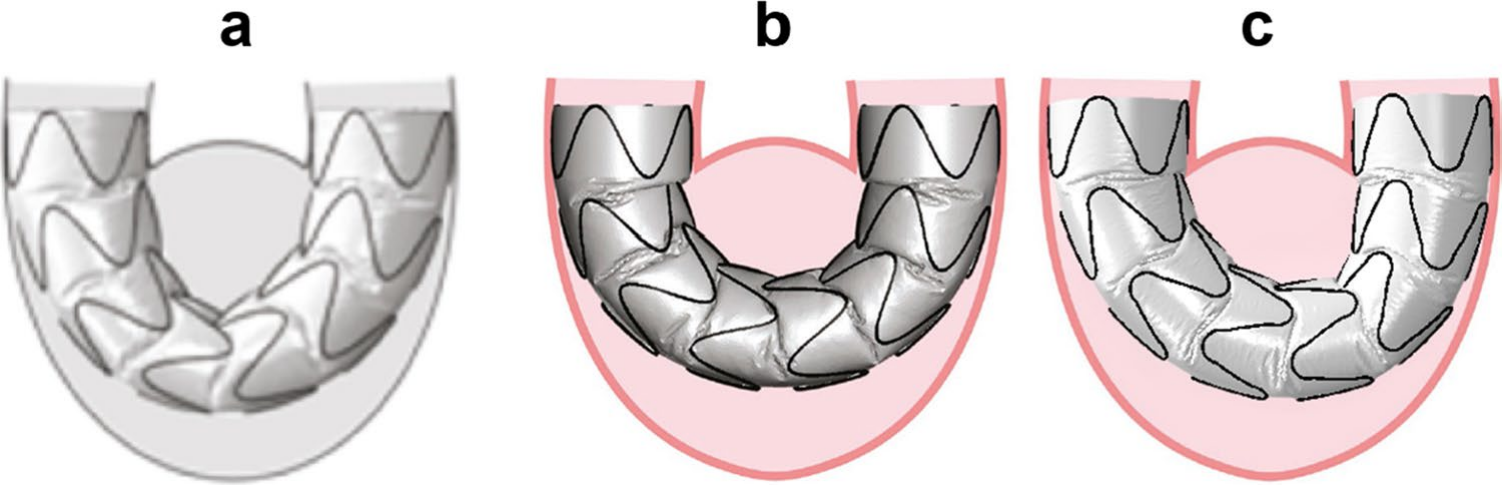
Comparison between the overall behavior of Zenith-LP in **a** Demanget et al. [14], **b** Dalbosco et al. [22], and **c** current work

To demonstrate a reasonable accuracy in predicting the bending behavior of the Zenith-LP SG (computational characteristics can be found in Table 1) after intraluminal pressurization, a comparison between the predicted results in Demanget et al. [16] (Fig. 3a), Dalbosco et al. [19] (Fig. 3b), and in the present work (Fig. 3c) is depicted in Fig. 3. The overall behavior of all three SGs are relatively similar, in spite of in the suture modeling strategy and choice of elements for the stent and FEM software used.

**Fig. 4:**
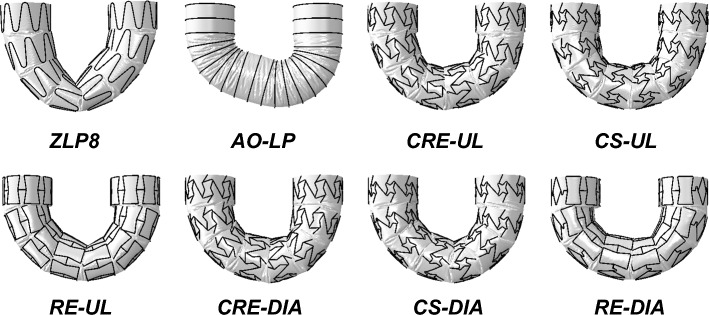
Deformed state of all SG candidates after intraluminal pressurization

**Fig. 5:**
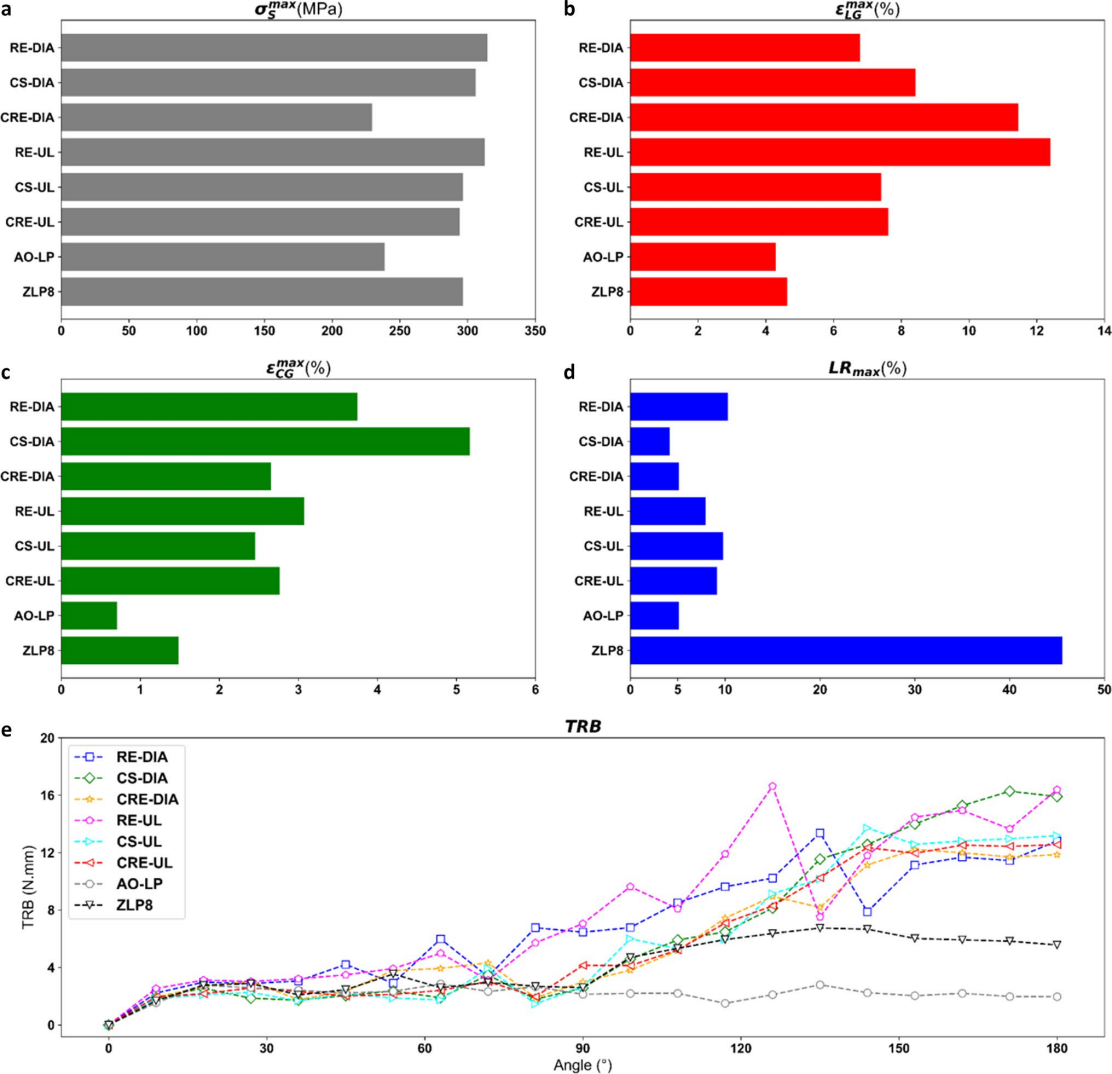
Compilation of assessment criteria for evaluating mechanical performance of SG candidates included in this study. **a** Maximal Von Mises Stress of stent $({\sigma }_{S}^{max})$, **b** maximal longitudinal membrane strain ${\varepsilon }_{LG}^{max}$, **c** maximal circumferential membrane strain ${\varepsilon }_{CG}^{max}$*,* **d** maximal luminal reduction rate $({LR}_{max})$ of SG candidates are all recorded post-intraluminal pressurization and **e** TRB vs bending angle curves for all SG candidates

Next, the results for all SG candidates are presented in terms of their global deformation properties in Fig. 4, maximal LR, maximal stresses in the stent, maximal strains in the graft, and torque-bending angle behavior observed during their respective performances in Fig. 5.

### Global Deformation Properties

Overall, ZLP8 suffered a noticeable kink in its central portion, where the graft was pulled downwards with buckling of the central stents. Among the novel SG designs derived from auxetic unit cells or NUPR unit cells in Fig. 4, it is difficult to isolate any top candidate in comparison based on the global deformation attributes in Fig. 4, with no noticeable kinks.

### Maximal Stresses in Stents $({\sigma }_{S}^{max})$

Taking into consideration the maximal stresses presented in Fig. 5a, we can categorize the SG candidates into two distinct groups. Group 1 includes AO-LP and CRE-DIA, both of which exhibit ${\sigma }_{S}^{max}$ values less than 250 MPa. In contrast, Group 2 encompasses the remaining stents, which display ${\sigma }_{S}^{max}$ values falling within the range of 250 to 320 MPa.

### Maximal Strains in Graft $({\varepsilon }_{LG}^{max}$ and ${\varepsilon }_{CG}^{max})$

Among the candidates depicted in Fig. 5b and c, AO-LP and ZLP8 displayed the lowest strain characteristics. Specifically, they showed ${\varepsilon }_{LG}^{max}$ values less than 5% and ${\varepsilon }_{CG}^{max}$ values less than 2%. In contrast, RE-UL and CS-DIA recorded the highest maximal strains in Fig. 5b and c, with ${\varepsilon }_{LG}^{max}$ exceeding 12% and ${\varepsilon }_{CG}^{max}$ surpassing 5%. It's worth noting that both of these values fall within the critical strain limit for the graft fabric. As observed by Demanget et al. [16], a consistent observation across all SG candidates was that the peak fabric strains were most prominent on the inner curvature of the SG, particularly between the stent rings (stent spacing) or between individual stent peaks. This occurrence is influenced by the overlapping of stent struts in these specific areas.

### Maximal Luminal Reduction Rate $( {LR}_{max})$

Among the novel SG candidates originating from auxetic unit cells or NUPR unit cells, all exhibited a ${LR}_{max}$ magnitude below 12% in Fig. 5d, suggesting their suitability for clinical applications. Within this group, AO-LP, CRE-DIA, and CS-DIA emerged as the only candidates with ${LR}_{max}$ values below 6%. As expected with the observed kink in its central region, ZLP8 displayed the highest ${LR}_{max}$ magnitude among all SG candidates, approaching 45%, which is in proximity to the critical clinical threshold of 60%.

### Torque Required for Bending (TRB)

According to the torque-angle curves presented in Fig. 5e, AO-LP emerges as the most flexible SG candidate, as it exhibits a peak torque of less than 2 N mm. ZLP8 and CRE-DIA closely follow in terms of flexibility, with torque values of 5 N mm and 12 N mm, respectively. It's worth noting that AO-LP maintains a consistent and stable torque curve behavior throughout a 180° angulation, a distinctive characteristic when compared to the novel SG candidates derived from auxetic unit cells. The latter exhibit a relatively linear increase in torque magnitude in the range of 60° to 120°, as depicted in Fig. 5e. In the case of ZLP8 (Fig. 4), the formation of kinks in the central region is associated with the torque increase observed between 80° and 150° of angulation. The oscillation seen in the torque-angle curves of SG candidates derived from auxetic unit cells can be attributed to graft wrinkling when being pulled inward by the stents during bending after a 45° angulation.

### Crimp Test: Principal Strain and Force–Diameter Curves

**Fig. 6:**
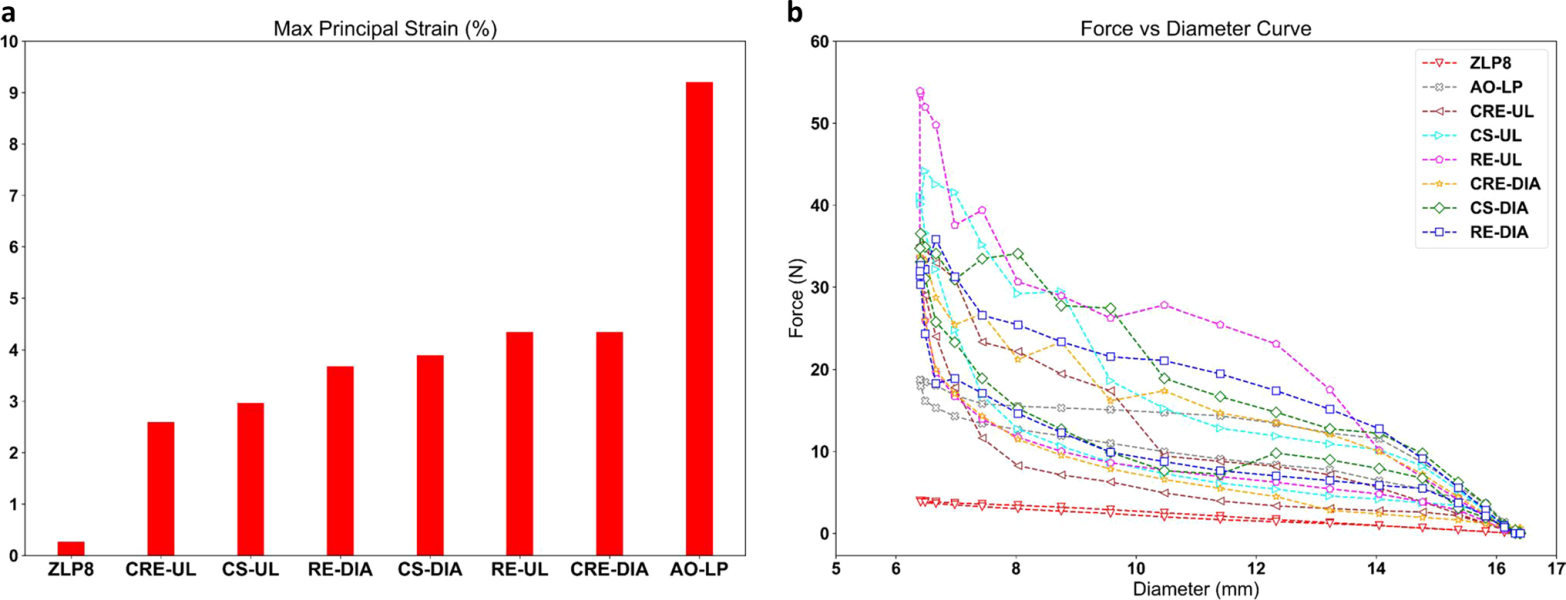
Compilation of assessment criteria for crimp test of SG candidates. The maximal principal strain at the final crimped stage (**a**) and radial force–diameter curves (**b**) of the novel SG candidates compared to ZLP8 SG

As depicted in Fig. 6a, it is evident that all stents originating from either auxetic unit cells or a combination of NUPR unit cells exhibit maximal principal strain values within the range of 2.5–4.5%. These values fall significantly below the critical limit of 12% for Nitinol. Figure 6b shows that all these stents, derived from auxetic unit cells or NUPR unit cell combinations, produce a peak radial force within the range of 32–53 N, which is over eight times greater than that of ZLP8 (4 N). AO-LP has been reported to possess the highest crimping strain magnitude among all other stent designs considered in this study.

## Discussion

Few works have explored the influence of stent design on SG flexibility in recent literature. A different approach examining the link between SG microstructure and flexibility based on its effect on stent graft-induced new entry (SINE) after thoracic endovascular aortic repair (TEVAR) was conducted by Guan et al. [23], where stent spacing, apex angle, and strut configuration (Z- and M-shaped) were selected as structural parameters to investigate their effect on bending and spring-back forces. According to [23], optimizing SG flexibility can be achieved through enhanced stent spacing, a larger stent apex angle, and usage of z-stent shape over m-stent shape. Yan et al. [24] suggested non-equal-strut stent hoops to improve radial support force and longitudinal flexibility, which were proven through numerical and experimental testing. In another study by Liu et al. [20], reducing stent height and graft thickness had the most significant impact on enhancing global flexibility for SGs used in TEVAR. Moreover, their orthogonal design study revealed that wire diameter is the most critical factor for improving local flexibility, affecting the sealing zone between the SG and aortic arch. Noting the lack of research on the utilization of auxetic stent design for SG optimization, we decided to investigate the potential of auxetic SGs for adverse angulated settings faced in EVAR.

Following the demonstration of the potential of three auxetic stent candidates (RE, CRE, and CS) to enhance conventional SG designs in our prior study [17], this paper showcases the performance of SGs derived from auxetic unit cells in an extremely tortuous iliac aneurysm characterized by a 180° curvature. Drawing inspiration from the research of Han and Lu [24], we introduced stents featuring NUPR that combine auxetic unit cells with a diamond unit cell [17] into our study. This combination resulted in the creation of SGs from hybrid auxetic unit cells, namely RE-DIA, CRE-DIA, and CS-DIA. For conciseness, when we refer to “novel SGs” in the subsequent discussion, we are addressing the entire collection of these new SG designs (RE-UL, CRE-UL, CS-UL, RE-DIA, CRE-DIA, CS-DIA). To replicate the in-vivo deformations that such novel SGs might encounter after deployment in a highly tortuous iliac aneurysm, we adhered to the modeling approach outlined by Demanget et al. [16] and applied specific boundary conditions.

As illustrated in Fig. 4, the novel SGs exhibited a noteworthy absence of kinks, displaying uniform distortions consistently along their entire length in their deformed states. This aligns with the findings of Demanget et al. [14, 16], who observed that SGs employing circular, spiral, or Z-spiral stents showcased enhanced flexibility compared to standard Z-stented SGs like Zenith-LP. This distinction is evident in Fig. 4, which depicts a noticeable kink in the central area of ZLP8. Importantly, none of the SGs in our study displayed stress magnitudes exceeding 390 MPa, as depicted in Fig. 5a. This indicates that the nitinol stents retained their elastic properties throughout the simulation, suggesting that the novel SGs are unlikely to experience stent fractures, as they did not approach the ultimate strength of nitinol (1172 MPa). Demanget et al. [16] also noted that none of the SGs in their research exhibited graft strains surpassing 20% (which is the ultimate strain limit for PET), a finding consistent with our study, as shown in Fig. 5b, c. This reaffirms that the novel SGs exhibit satisfactory reliability in terms of both the stent and graft components when subjected to high tortuosity conditions.

In the context of flexibility, all the novel SGs displayed values considerably below the critical luminal reduction threshold of 60%, as evident in Fig. 5d. This substantiates the suitability of these SGs for EVAR applications, particularly in situations involving intricate iliac angulations with high tortuosity. Notably, AO-LP (a circular-stented SG) exhibited superior flexibility, a characteristic consistent with the findings reported in Demanget et al. [14], as indicated by the torque vs. angle curves in Fig. 5e. As an additional evaluation, we assessed the radial properties of the novel SG candidates by comparing them to ZLP8 through a crimp test involving a single stent ring, the results of which are presented in Fig. 6. All the novel stents exhibited strain magnitudes at the crimped diameter (corresponding to a 60% reduction in stent diameter, Fig. 6a) that fall within an acceptable range. This signifies a low risk of stent rupture during the crimping process of these candidates into the delivery sheath. Furthermore, all the novel stents demonstrated higher peak radial force attributes compared to the ZLP8 stent, as illustrated in Fig. 6b. This indicates their capability to meet or even surpass the radial requirements of a commercial stent design commonly used in EVAR applications. It's noteworthy to mention that De Bock et al. [25] have emphasized that a SG design with enhanced radial strength at the sealing fixation zone should exhibit lower radial force at the suprarenal fixation zone, which could contribute to improved long-term success.

Kwiecinski et al. [26] documented an oversizing range of 26–35% for commercially available SGs used in chimney thoracic endovascular aortic repair (ch-TEVAR). In EVAR, SG iliac limb kinking has been identified as a common cause of limb occlusion complications, and excessive SG oversizing is a potential contributing risk factor [27]. The novel SGs hold promise as a potential solution to mitigate this complication. Their higher radial force, as demonstrated in the crimp tests, obviates the need for excessive oversizing to meet the clinical demands for radial strength. Moreover, these novel SG stents exhibit no kinks even at high iliac artery angulations, as evidenced in this study. Considering that stent designs characterized by spiral or helical-spiral configurations (similar to Aorfix) are known to experience reduced longitudinal support [28], we propose that SGs constructed from auxetic unit cells could offer a superior solution in situations requiring enhanced longitudinal support, as measured by increased fixation/drag forces, along with improved radial stiffness, which leads to enhanced sealing against the aortic wall. This can be achieved while still maintaining flexibility and minimizing luminal reduction rates.

## Limitations

A key limitation considering the methodology replicated in the current study is the modeling of sutures to link the stent rings to the graft membrane in commercial SGs. Sutures were represented by a continuous tie constraint in Abaqus, which estimates the bond between the stent and graft to reduce the complexity of the contact between the stent and graft in present work and thereby diminishes computational expense. It is to be noted that computational methodologies involving the tie constraint simplification have successfully predicted SG deployment in aortic aneurysms [29]. Recent works, such as Acosta Santamaria et al. [30], replicated SG links as specified tie conditions at specific suture sections as per clinical feedback, and Dalbosco et al. [19] modeled sutures as rigid beams at specific points to estimate suture forces (reaction forces acting on beam elements), which are linked to SG fatigue. Dalbosco et al. [19] compared numerical results to experimental data in scientific literature to demonstrate higher accuracy in predicting the prevalence of suture detachment using their proposed methodology. Future studies are recommended to follow the methodology proposed by Dalbosco et al. [19] to identify the best suture patterns that would enable novel SG’s to perform at their best capabilities and avoid suture detachment.

A secondary limitation is the absence of stent pre-stress [29, 31] in present work, which plays an important role in influencing SG global behavior. Since the stress-free stent diameter (16 mm) is not much larger than the stress-free graft diameter (15.644 mm), we excluded stent pre-stress from modeling the mechanical behavior of all SG models in this study, considering that we do not have any experimental data to validate the effect of stent pre-deformation and the exceptional computational effort to include this in SG simulations.

One potential implication of disregarding stent pre-stress is the risk of overestimating material properties, which may result in a stiffer or less flexible representation of the SG in simulation results.

To determine the extent of error in the maximal stresses in the stent reported in this study, we monitored the maximal stresses in the stent for all candidates during the crimp test when the stent diameter was reduced to a value close to the stress-free graft diameter. We observed that the maximal Von Mises stress at a diameter of 15.65 mm was in the range of 10–12 MPa for all crimp test candidates. Based on this observation, we can estimate that the error in maximal stress in the stent caused by residual stresses due to stent pre-deformation can be quantified to be approximately ± 12 MPa. Taking into account the error estimate in the reported stress magnitudes for the SG candidates in this study, we can affirm that none of the SGs exceed the stress threshold necessary to trigger the forward martensite transformation, which is set at 390 MPa. This confirms their consistent linear elastic behavior during the simulations. Furthermore, the stress levels in these SG candidates do not approach the ultimate tensile stress range, which falls between 827 and 1172 MPa as specified in the material properties of Nitinol, as reported in Demanget et al. [16]. This effectively reduces the likelihood of stent fractures being present for these SG candidates in highly tortuous settings.

A significant limitation concerns the lack of experimental validation. The objective was to use numerical simulations to explore new auxetic designs to optimize the response of SGs to severe bending. The numerical model used to run these simulations has been validated with rigorous experimental testing [15]. The next step is to fabricate auxetic SG prototypes to confirm their performance in bending loading conditions.

## Conclusions

In conclusion, the novel SG candidates displayed promising mechanical performance for EVAR applications in highly tortuous iliac aneurysms, even with angulations up to 180°. They exemplify strong reliability characteristics in terms of maximal stress on stents and maximal graft strain, showing a low probability of stent fracture and graft tear, respectively, in such aneurysms. Overall, no specific advantage has been identified for a SG derived from NUPR unit cells (CRE-DIA, CS-DIA, RE-DIA) over a SG derived from standard auxetic unit cells (CRE-UL,CS-UL,RE-UL) based on their mechanical performance. Most importantly, all novel SGs exhibit lower luminal reduction rate values and higher radial force magnitude in contrast to a conventional Z-stented SG. Thus, a future investigation of their mechanical behavior after deployment in an idealized AAA [21] will be conducted to examine their interaction with the artery wall.

### Supplementary Information

Below is the link to the electronic supplementary material.

Supplementary file1 (DOCX 282 KB)

## Abbreviations

SG: Stent-graft
Zenith LP: Zenith Low Profile
FEA: Finite element analysis
RE: Re-entrant
EVAR: Endovascular Aneurysm Repair
CRE: Chiral-re-entrant hybrid
CS: Chiral-star hybrid
RE-UL: Re-entrant unlinked
CRE-UL: Chiral-re-entrant unlinked
CS-UL: Chiral-star unlinked
CRE-DIA: Chiral-re-entrant-diamond hybrid
CS-DIA: Chiral-star-diamond hybrid
RE-DIA: Re-entrant-diamond hybrid
PET: Polyethylene terephthalate
ZLP8: Zenith Low Profile 8 peaks
NUPR: Non-uniform Poissons’ ratio
${\sigma }_{S}^{max}$: Maximal Von Mises Stress in Stent
${LR}_{max}$: Maximal luminal reduction rate
${\varepsilon }_{CG}$: Circumferential logarithmic strain in graft
${\varepsilon }_{LG}$: Longitudinal logarithmic strain in graft
AO-LP: Aorfix Low Profile

## References

[1] Parodi JC, Palmaz JC, Barone HD (1991). Transfemoral intraluminal graft implantation for abdominal aortic aneurysms. Ann. Vasc. Surg..

[2] Bae M, Chung SW, Lee CW, Song S, Kim E, Kim CW (2017). A comparative study of abdominal aortic aneurysm: endovascular aneurysm repair versus open repair. Korean J. Thorac Cardiovasc. Surg..

[3] Patel R, Sweeting MJ, Powell JT, Greenhalgh RM (2016). Endovascular versus open repair of abdominal aortic aneurysm in 15-years’ follow-up of the UK endovascular aneurysm repair trial 1 (EVAR trial 1): a randomised controlled trial. Lancet.

[4] Columbo JA, Ramkumar N, Martinez-Camblor P, Kang R, Suckow BD, O’Malley AJ, Sedrakyan A, Goodney PP (2020). Five-year reintervention after endovascular abdominal aortic aneurysm repair in the Vascular Quality Initiative. J. Vasc. Surg..

[5] Bryce Y, Rogoff P, Romanelli D, Reichle R (2015). Endovascular repair of abdominal aortic aneurysms: vascular anatomy, device selection, procedure, and procedure-specific complications. RadioGraphics.

[6] Cao P, Verzini F, Zannetti S, De Rango P, Parlani G, Lupattelli L, Maselli A (2002). Device migration after endoluminal abdominal aortic aneurysm repair: analysis of 113 cases with a minimum follow-up period of 2 years. J. Vasc. Surg..

[7] Zarins CK, Arko FR, Crabtree T, Bloch DA, Ouriel K, Allen RC, White RA (2004). Explant analysis of AneuRx stent grafts: relationship between structural findings and clinical outcome. J. Vasc. Surg..

[8] Krishnamoorthi H, Jeon-Slaughter H, Wall A, Banerjee S, Ramanan B, Timaran C, Modrall JG, Tsai S (2018). Rate of secondary intervention after open versus endovascular abdominal aortic aneurysm repair. J. Surg. Res..

[9] Umscheid T, Stelter WJ (1999). Time-related alterations in shape, position, and structure of self-expanding, modular aortic stent-grafts: a 4-year single-center follow-up. J. Endovasc. Surg..

[10] Carroccio A, Faries PL, Morrissey NJ, Teodorescu V, Burks JA, Gravereaux EC, Hollier LH, Marin ML (2002). Predicting iliac limb occlusions after bifurcated aortic stent grafting: anatomic and device-related causes. J. Vasc. Surg..

[11] Albertini J-N, DeMasi M-A, Macierewicz J, El Idrissi R, Hopkinson BR, Clément C, Branchereau A (2005). aorfix stent graft for abdominal aortic aneurysms reduces the risk of proximal type 1 endoleak in angulated necks: Bench-Test Study. Vascular.

[12] Weale AR, Balasubramaniam K, Hardman J, Horrocks M (2010). Use of the AorfixTM stent graft in patients with tortuous iliac anatomy. J. Cardiovasc. Surg. (Torino).

[13] Kleinstreuer C, Li Z, Basciano CA, Seelecke S, Farber MA (2008). Computational mechanics of Nitinol stent grafts. J. Biomech..

[14] Demanget N, Avril S, Badel P, Orgéas L, Geindreau C, Albertini J-N, Favre J-P (2012). Computational comparison of the bending behavior of aortic stent-grafts. J. Mech. Behav. Biomed. Mater..

[15] Demanget N, Latil P, Orgéas L, Badel P, Avril S, Geindreau C, Albertini J-N, Favre J-P (2012). Severe bending of two aortic stent-grafts: an experimental and numerical mechanical analysis. Ann. Biomed. Eng..

[16] Demanget N, Duprey A, Badel P, Orgéas L, Avril S, Geindreau C, Albertini J-N, Favre J-P (2013). Finite element analysis of the mechanical performances of 8 marketed aortic stent-grafts. J. Endovasc. Ther..

[17] Vellaparambil R, Han W-S, Di Giovanni P, Avril S (2023). Potential of auxetic designs in endovascular aortic repair: a computational study of their mechanical performance. J. Mech. Behav. Biomed. Mater..

[18] Perrin D, Demanget N, Badel P, Avril S, Orgéas L, Geindreau C, Albertini J-N (2015). Deployment of stent grafts in curved aneurysmal arteries: toward a predictive numerical tool. Int. J. Numer. Meth. Biomed. Eng..

[19] Dalbosco M, Roesler CRM, Silveira PG, Fancello EA (2020). Numerical study on the effect of stent shape on suture forces in stent-grafts. J. Mech. Behav. Biomed. Mater..

[20] Liu Z, Wu L, Yang J, Cui F, Ho P, Wang L, Dong J, Chen G (2021). Thoracic aorta stent grafts design in terms of biomechanical investigations into flexibility. Math. Biosci. Eng..

[21] Han Y, Lu W (2018). Optimizing the deformation behavior of stent with nonuniform Poisson’s ratio distribution for curved artery. Journal of the Mechanical Behavior of Biomedical Materials.

[22] Zenith Flex®. AUI AAA Endovascular Graft with the Z-Trak™ Introduction System Instructions for use. https://ifu.cookmedical.com/ifuPub/ReadFile?fileName=T_ZAUI_REV1.PDF.

[23] Guan Y, Lin J, Dong Z, Wang L (2018). Comparative study of the effect of structural parameters on the flexibility of endovascular stent grafts. Adv. Mater. Sci. Eng..

[24] Yan S, Song C, Si Y, Zhao Y (2022). Design of non-equal-strut stent hoops for structural optimization of thoracic aortic stent-grafts. Minim. Invas. Ther. Allied Technol..

[25] De Bock S, Iannaccone F, De Beule M, Van Loo D, Vermassen F, Verhegghe B, Segers P (2013). Filling the void: a coalescent numerical and experimental technique to determine aortic stent graft mechanics. J. Biomech..

[26] Kwiecinski J, Cheng CP, Uberoi R, Hadi M, Hempel P, Degel C, You Z (2021). Thoracic aortic parallel stent-graft behaviour when subjected to radial loading. J. Mech. Behav. Biomed. Mater..

[27] Coelho A, Nogueira C, Lobo M, Gouveia R, Campos J, Augusto R, Coelho N, Semião AC, Ribeiro JP, Canedo A (2019). Impact of post-EVAR graft limb kinking in EVAR limb occlusion: aetiology, early diagnosis, and management. Eur. J. Vasc. Endovasc. Surg..

[28] Stoeckel D, Bonsignore C, Duda S (2002). A survey of stent designs. Minim. Invasive Ther. Allied Technol..

[29] Perrin D, Badel P, Orgéas L, Geindreau C, Dumenil A, Albertini J-N, Avril S (2015). Patient-specific numerical simulation of stent-graft deployment: validation on three clinical cases. J. Biomech..

[30] Acosta Santamaría VA, Daniel G, Perrin D, Albertini JN, Rosset E, Avril S (2018). Model reduction methodology for computational simulations of endovascular repair. Comput. Methods Biomech. Biomed. Eng..

[31] Ramella A, Migliavacca F, Rodriguez Matas JF, Heim F, Dedola F, Marconi S, Conti M, Allievi S, Mandigers TJ, Bissacco D, Domanin M, Trimarchi S, Luraghi G (2022). Validation and verification of high-fidelity simulations of thoracic stent-graft implantation. Ann. Biomed. Eng..

