# Supplementary material for: Computational Comparison of the Mechanical Behavior of Aortic Stent-Grafts Derived from Auxetic Unit Cells
Source: Cardiovasc Eng Technol. 2023 Dec 18;15(2):199–210. doi: 10.1007/s13239-023-00706-x (PMC11149442; doi:10.1007/s13239-023-00706-x)
Supplement: Supplementary file 1 — Supplementary file1 (DOCX 282 KB) [file 13239_2023_706_MOESM1_ESM.docx]

Appendix / Supplementary Information (SI)

Article name: **Computational comparison of the mechanical behaviour of aortic stent-grafts derived from auxetic unit cells**

Journal name: Cardiovascular Engineering and Technology

Author Names:

Rahul Vellaparambil ^a,b^, Woo-Suck Han ^a^, Pierluigi Di Giovanni ^b^, Stéphane Avril ^a^

Affliations:

^a^ Centre CIS, Mines Saint-Etienne, Université Jean Monnet Saint-Etienne, INSERM, SAINBIOSE U1059, F-42023 Saint-Etienne, France

^b^Research and Development Department, HSL S.R.L, Trento, Italy

| **Parameter** | **Values** |
| --- | --- |
| Austenite elasticity (MPa) | 40000 |
| Austenite Poisson’s ratio | 0.46 |
| Martensite elasticity (MPa) | 18554 |
| Martensite Poisson’s ratio | 0.46 |
| Transformation strain | 0.04 |
| Start of transformation loading (MPa) | 390 |
| End of transformation loading (MPa) | 425 |
| Ultimate tensile strength (MPa) | 827-1172 |

*Table S1: Constituent parameters for Nitinol model [14]*

| **Parameter** | **Values** |
| --- | --- |
| Longitudinal elastic modulus (MPa) | 225 |
| Circumferential elastic modulus (MPa) | 1000 |
| Poisson ratio | 0.2 |
| In-plane shear modulus (MPa) | 3.6 |
| Longitudinal ultimate strain | 0.23 |
| Circumferential ultimate strain | 0.18 |
| Longitudinal bending stiffness (x 10^-4^ N.mm) | 4 |
| Circumferential bending stiffness (x 10^-4^ N.mm) | 18 |

*Table S2: Constituent parameters for PET graft [14]*

| **Parameter** | **Values** |
| --- | --- |
| Graft radius | 7.822 |
| Graft length | 92.9 |
| Graft Thickness | 0.08 |
| Stent Height (mm) | 8.6 |
| Stent Wire radius (mm) | 0.17 |
| Peaks per circumference of stent | 4 |
| Number of stent rings along SG length | 8 |

*Table S3: Dimensional Attributes of Zenith-LP SG [14]*

| **Parameter** | **Values** |
| --- | --- |
| Graft radius | 7.867 |
| Graft length | 88.0 |
| Graft Thickness | 0.08 |
| Stent Wire radius (mm) | 0.125 |
| Number of stent rings along SG length | 20 |

*Table S4: Dimensional Attributes of Aorfix spiral-stented SG [14]*


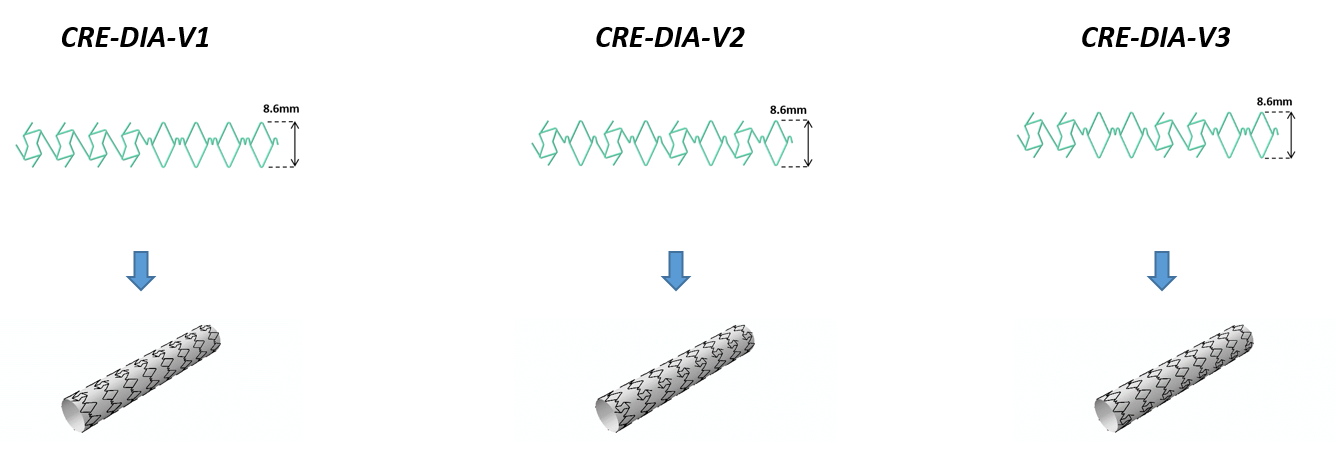


**Fig. S1**: Illustration of three CRE-DIA SG variants utilized in an optimization study


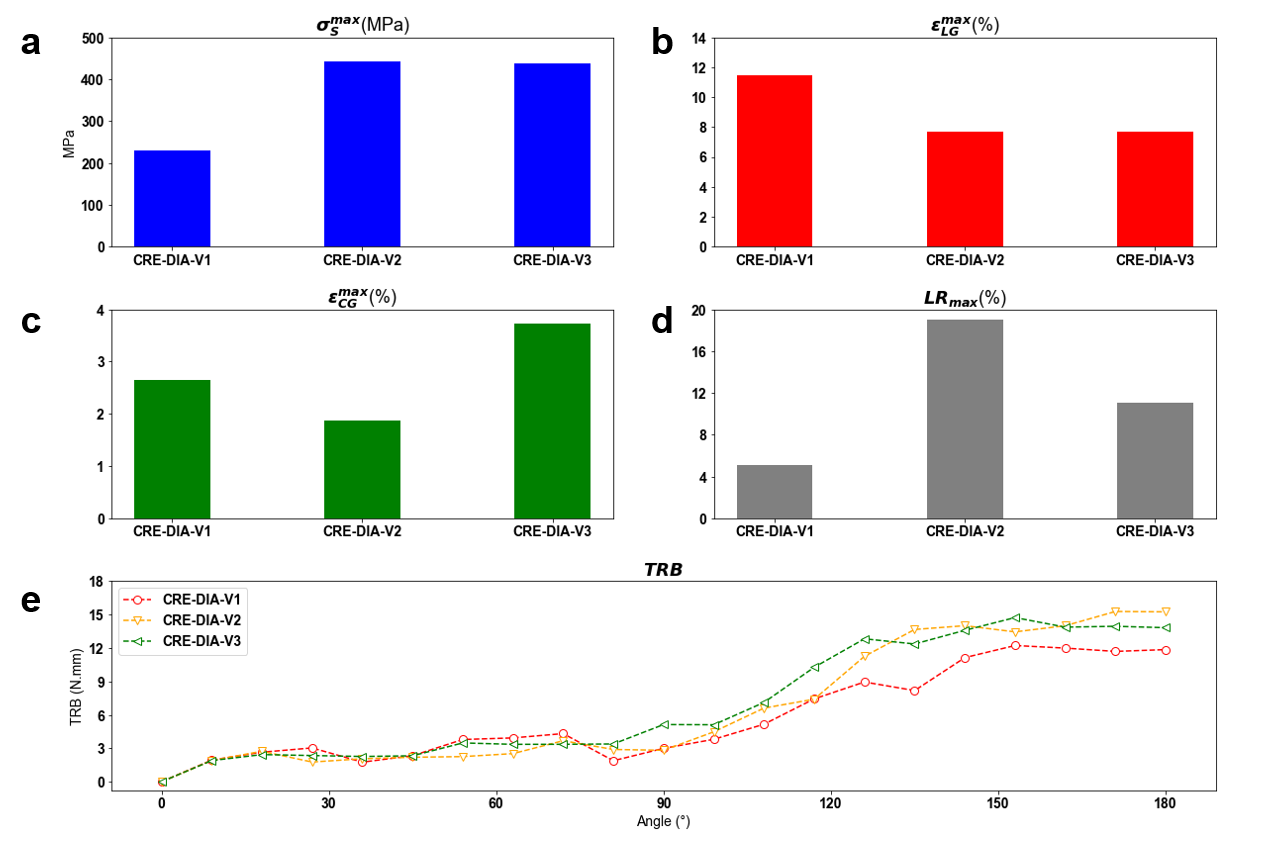


**Fig. S2**: *Compilation of assessment criteria for evaluating mechanical performance of three CRE-DIA variants included in optimization study. a: Maximal Von Mises Stress of stent(* $\sigma_{S}^{max}$*), b: maximal longitudinal membrane strain* $\varepsilon_{LG}^{max}$*, c: maximal circumferential membrane strain* $\varepsilon_{CG}^{max}$*, d: maximal luminal reduction rate (* ${LR}_{max})$ *of CRE-DIA variants are all recorded post-intraluminal pressurization and e: Torque required for bending (TRB) vs bending angle curves for all CRE-DIA variants.*

In order to obtain the best arrangement of a non-uniform Poisson’s ratio (NUPR) stent design, we generated three variants of CRE-DIA stent design as observed in figure S1 and tested their mechanical response to a 180° U-bend and intraluminal pressurization. CRE-DIA-V1 emerges as the top-performing variant among the three variants of CRE-DIA design as it demonstrates the lowest $\sigma_{S}^{max}$ and ${LR}_{max}$ magnitudes as noted in figure S2a and figure S2d respectively. Maximal graft strain magnitude in both directions ( $\varepsilon_{CG}^{max}$ , $\varepsilon_{lG}^{max} )$ for CRE-DIA-V1 did not exceed the critical limit of 20% for PET as depicted in figure S2b & figure S2c respectively. Based on this evidence, we used the same arrangement of auxetic and diamond unit cells in CRE-DIA-V1 to generate CS-DIA and RE-DIA SGs with CS & RE auxetic unit cells respectively, as depicted in figure 1.
